# Circulating Microbial Products and Acute Phase Proteins as Markers of Pathogenesis in Lymphatic Filarial Disease

**DOI:** 10.1371/journal.ppat.1002749

**Published:** 2012-06-07

**Authors:** R. Anuradha, P. Jovvian George, N. Pavan Kumar, Michael P. Fay, V. Kumaraswami, Thomas B. Nutman, Subash Babu

**Affiliations:** 1 National Institutes of Health, International Center for Excellence in Research, Chennai, India; 2 Biostatistics Research Branch, National Institute of Allergy and Infectious Diseases, National Institutes of Health, Bethesda, Maryland, United States of America; 3 Tuberculosis Research Center, Chennai, India; 4 Laboratory of Parasitic Diseases, National Institute of Allergy and Infectious Diseases, National Institutes of Health, Bethesda, Maryland, United States of America; 5 SAIC-Frederick, Inc., NCI-Frederick, Frederick, Maryland, United States of America; Washington University, United States of America

## Abstract

Lymphatic filariasis can be associated with development of serious pathology in the form of lymphedema, hydrocele, and elephantiasis in a subset of infected patients. Dysregulated host inflammatory responses leading to systemic immune activation are thought to play a central role in filarial disease pathogenesis. We measured the plasma levels of microbial translocation markers, acute phase proteins, and inflammatory cytokines in individuals with chronic filarial pathology with (CP Ag+) or without (CP Ag−) active infection; with clinically asymptomatic infections (INF); and in those without infection (endemic normal [EN]). Comparisons between the two actively infected groups (CP Ag+ compared to INF) and those without active infection (CP Ag− compared to EN) were used preliminarily to identify markers of pathogenesis. Thereafter, we tested for group effects among all the four groups using linear models on the log transformed responses of the markers. Our data suggest that circulating levels of microbial translocation products (lipopolysaccharide and LPS-binding protein), acute phase proteins (haptoglobin and serum amyloid protein-A), and inflammatory cytokines (IL-1β, IL-12, and TNF-α) are associated with pathogenesis of disease in lymphatic filarial infection and implicate an important role for circulating microbial products and acute phase proteins.

## Author Summary

Lymphatic filariasis afflicts over 120 million people worldwide. While the infection is mostly clinically asymptomatic, approximately 40 million people suffer from overt, morbid clinical pathology, characterized by swelling of the scrotal area and lower limbs (hydrocele and lymphedema). Host immunologic factors that influence the pathogenesis of disease in these individuals are not completely understood. Circulating microbial products such as LPS and markers associated with microbial translocation have been shown to play an important role in disease pathogenesis of certain infections like HIV. Similarly, proteins associated with the acute phase response and related cytokines also play an important role in pathogenesis. We have attempted to elucidate the role of the above mentioned factors in disease pathogenesis by comparing the plasma levels of the various markers in four groups of individuals: chronic pathology individuals with or without active filarial infection, asymptomatic, filarial infected individuals and uninfected, endemic normal individuals. We show that circulating levels of LPS, acute phase proteins and certain cytokines are significantly elevated in filarial disease with active infection but not in the other groups indicating that filarial infection induced increased production of these factors correlated with the development of filarial lymphatic pathology.

## Introduction

Although two-thirds of the 120 million people infected with *Wuchereria bancrofti*—the major causative agent of human lymphatic filariasis—have subclinical infections, ∼40 million have lymphedema and/or other pathologic manifestations including hydroceles (and other forms of urogenital disease), episodic adenolymphangitis, tropical pulmonary eosinophilia, lymphedema, and (in its most severe form) elephantiasis [1]. It is assumed that repeated episodes of acute inflammation can lead to development of serious disfigurement in the face of compromised lymphatics [2], although many other factors that contribute to the pathology associated with lymphatic filariasis are largely unknown. Typically in *Wuchereria* or *Brugia* infections, disease manifests years after exposure, while clinically asymptomatic infection is not only more common but can occur at a relatively young age [3], [4].

Lymphatic filarial disease is felt to be a reflection of both localized and systemic immunologic and inflammatory responses mediated by pro-inflammatory cytokines and chemokines [5], [6]. Although some of the pathological changes can likely be initiated by Wolbachia- or parasite-encoded endotoxin-like substances and/or secondary bacterial or fungal infections [1], [7], chronic parasite-induced immune activation is a salient feature of filarial disease. Indeed, increased frequencies of activated T cells [8], increased parasite antigen-driven Th1 and Th17 cytokine production [6], increased expression of Toll-like and NOD-like receptors [6], and enhanced TLR signaling through TLR ligand stimulation [5] have all been described when comparisons are made between patients with subclinical infection and those with filarial lymphedema and/or elephantiasis. Moreover, innate immune responses also play a prominent role in development of pathology, as evidenced by the occurrence of lymphatic damage in animal models of filarial infection lacking an adaptive immune system [9].

Persistent immune activation is associated with elevations of circulating microbial products, acute phase proteins, and the so-called microbial translocation molecules [10]. Translocation of microbial products from the lumen of the intestine into the periphery is thought to contribute to induction of inflammation by stimulating immune effector cells directly through their pattern recognition receptors [11]; however, intra- and peri-lymphatic damage—an underlying feature of filarial disease [12]—might also contribute to the presence of microbial translocation products in the bloodstream. In addition, chronic immune activation that often accompanies infectious processes [13] is associated with development of an acute phase response and the presence of markers of inflammation in plasma. Moreover, increased serum levels of proinflammatory cytokines and chemokines are commonly associated with progressive immune activation.

In this study, we have delineated the role of many of the known markers of inflammation and lymphatic damage that reflect the dysregulated (or unchecked) responses related to development of disease with the lymphatic-dwelling filariae (*Wuchereria bancrofti* and *Brugia malayi*). Our data suggest that circulating (systemic) microbial products, acute phase proteins, and pro-inflammatory cytokines reflect the localized (and ongoing) chronic immune activation that underlies the pathogenesis of disease in lymphatic filariasis.

## Materials and Methods

### Study population

We studied a group of 91 individuals with filarial lymphedema without active filarial infection (hereafter CP Ag−), 28 individuals with filarial lymphedema with active filarial infection (hereafter CP Ag+), 98 asymptomatic or subclinical, infected individuals (hereafter INF), and 82 uninfected, endemic normal individuals (hereafter EN) in an area endemic for lymphatic filariasis in Tamil Nadu, South India (table 1). Diagnosis of active filarial infection was performed by measuring circulating filarial antigen levels by both the ICT filarial antigen test (Binax, Portland, ME, USA) and the TropBio Og4C3 enzyme-linked immunosorbent assay (ELISA) (Trop Bio Pty. Ltd, Townsville, Queensland, Australia). All the CP Ag− individuals had undergone treatment with repeated doses of diethylcarbamazine (DEC). None of the CP Ag+ individuals had received any DEC treatment but were administered DEC following the blood draw. All of the CP individuals had early stage lymphedema (Grades 1 and 2) only and individuals with concurrent overt and active bacterial infection were excluded from the study. All individuals were examined as part of a clinical protocol approved by Institutional Review Boards of both the National Institute of Allergy and Infectious Diseases and the Tuberculosis Research Center (NCT00375583 and NCT00001230), and informed written consent was obtained from all participants.

**Table 1:** Characteristics of the study population.

	Endemic Normal	Infected	Chronic Pathology	Chronic Pathology
	(EN) (*n* = 82)	(INF) (*n* = 98)	(CP Ag+) (*n* = 28)	(CPAg−) (*n* = 91)
Age	26 (20–50)	36 (15–73)	44 (18–69)	38 (17–70)
Sex M/F	44/38	52/46	19/9	47/44
CFA* (IU)	<32	3126 (136–32000)	1606 (464–8996)	<32
Pathology Stages 1/2	Nil	Nil	12/16	42/49

*: CFA values are determined by the Og4C3 ELISA and 32 IU was the threshold of detection in the assay.

### Microbial translocation assays

To inactivate plasma proteins, plasma samples were heated to 75°C for 5 min. Lipopolysaccharide (LPS) levels were measured using a limulus amebocyte lysate assay (Cell Sciences Hycult Biotech, Canton, MA, USA) according to the manufacturer's protocol. Commercially available ELISA kits were used to measure plasma levels of LPS- binding protein (LBP), endotoxin core antibodies IgG (EndoCAb) (Cell Sciences Hycult Biotech), and soluble CD14 (sCD14) (R&D Systems, Minneapolis, MN, USA).

### Acute phase proteins

Plasma levels of C-reactive protein (CRP), haptoglobin, serum amyloid protein - A (SAA), and α-2 macroglobulin (α-2M) were measured using the Bioplex (Bio-Rad, Hercules, CA, USA) multiplex ELISA system according to the manufacturer's instructions.

### Cytokines

Plasma levels of cytokines, IL-1β, IL-6, IL-12, and TNF-α (Bio-Rad) were measured using the Bioplex multiplex ELISA system.

### Statistical analysis

Data analyses were performed using GraphPad PRISM (GraphPad Software, Inc., San Diego, CA, USA). Geometric means (GM) were used for measurements of central tendency. Preliminary statistical analysis was done using the non-parametric Mann-Whitney test. We then tested for group effects using linear models on the log transformed data. We used robust standard error with a recommended bias adjustment so that we need not assume that the error variance was the same for each group. We parameterized the 4 group effects using parameters for CP, infection, and CP by infection interaction. Since we tested these 3 parameters on 12 markers, we adjusted the p-values for multiple comparisons using Holm's adjustment. P-values in Table 2 are Holm's adjusted. We then built specific models using only the significant (when Holm's adjusted p-value<0.05) effects, and we present those effects and (unadjusted) 95% confidence intervals as fold-change (Table 3). We repeated the models after adding an effect for age (either as a continuous or a categorical variable). Linear models were done in R 2.14.0 using the sandwich R package. Correlations were calculated by the Spearman rank correlation test.

**Table 2:** Holm's adjusted p-values from Linear Models, No Age Effect, n = sample size.

	CP Effect	Infection Effect	Interaction Effect	n
LPS	0.6582	1.0000	<.0001	241
LBP	1.0000	1.0000	<.0001	232
EndoCAb	1.0000	1.0000	1.0000	249
sCD14	1.0000	1.0000	1.0000	289
CRP	<.0001	0.5797	1.0000	254
Haptoglobin	1.0000	1.0000	0.0001	210
SAA	1.0000	0.0357	0.8703	254
α-2-Macroglobulin	1.0000	0.6683	0.2362	251
IL-1β	1.0000	0.8534	0.0279	279
IL-6	0.0418	<.0001	0.5155	232
IL-12	1.0000	1.0000	<.0001	289
TNF-α	1.0000	0.0088	0.1485	278

**Table 3:** Fold-change for geometric mean of CP Ag+ group compared to others, with (unadjusted) 95% confidence intervals (CI).

	Fold change	Lower CI	Upper CI
LPS	54.27	19.11	154.14
LBP	0.012	0.004	0.043
Haptoglobin	3.14	1.96	5.01
IL-1β	3.15	1.60	6.19
IL-12	14.89	9.10	24.38

The heat map was constructed in JMP v8.0 (SAS, Carey, NC) and is based on relative expression for a given analyte as a function of the geometric mean value found in the endemic normal population.

## Results

### CP Ag+ exhibit elevated levels of LPS but lower levels of LBP compared with INF

**Figure 1:**
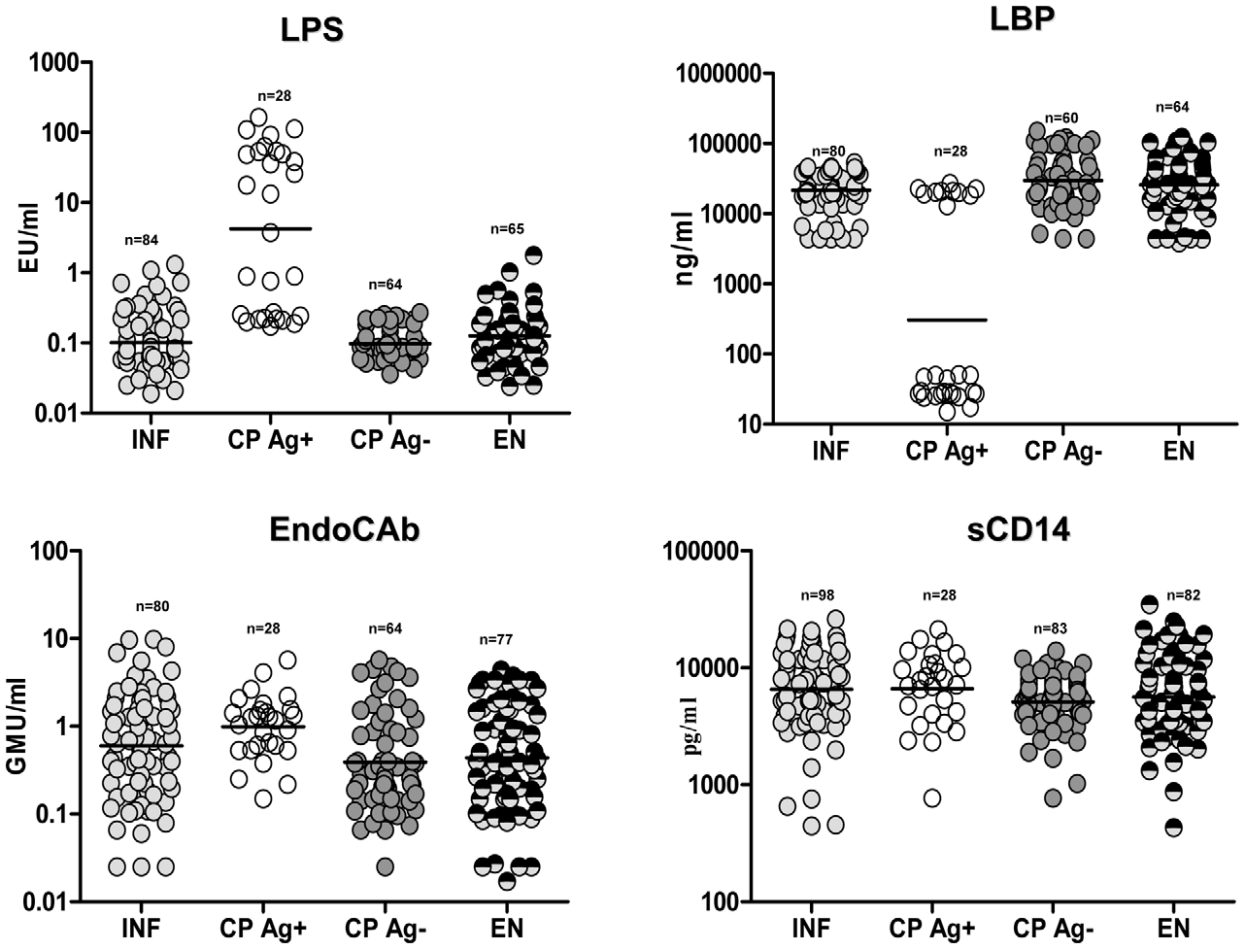
Filarial lymphedema is associated with elevated levels of LPS. Plasma levels of LPS, LBP, EndoCAb and sCD14 from asymptomatic infected [INF] individuals; filarial lymphedema individuals with active infection [CP Ag+]; filarial lymphedema individuals without active infection [CP Ag−] and endemic normal [EN] individuals were measured by ELISA and immunoassays. Data are shown as scatter plots with the bar representing the geometric mean.

To determine the association of microbial translocation and related markers with filarial lymphedema, we measured the plasma levels of LPS, LPB, EndoCAb, and sCD14 in CP Ag+, INF, CP Ag−, and EN. As shown in figure 1, CP Ag+ had significantly higher levels of LPS (GM of 4.24 EU/ml in CP Ag+ vs. 0.10 in INF; *P*<0.0001 by Mann-Whitney) but not sCD14 or EndoCAb in comparison to INF. Conversely, CP Ag+ had significantly lower levels of LBP (GM of 306.2 ng/ml in CP Ag+ vs. 21658 in INF; *P*<0.0001) in comparison to INF. However, no significant differences were observed in the levels of all four circulating microbial or related products between CP Ag− and EN. In addition, we consistently observed an inverse association between LPS and LBP levels in the CP Ag+ group (r2 = 0.862; *P*<0.0001). Thus, filarial lymphedema with active infection is characterized by elevated levels of circulating LPS.

### CP Ag+ exhibit elevated levels of CRP, haptoglobin, SAA, and α-2m compared with INF

**Figure 2:**
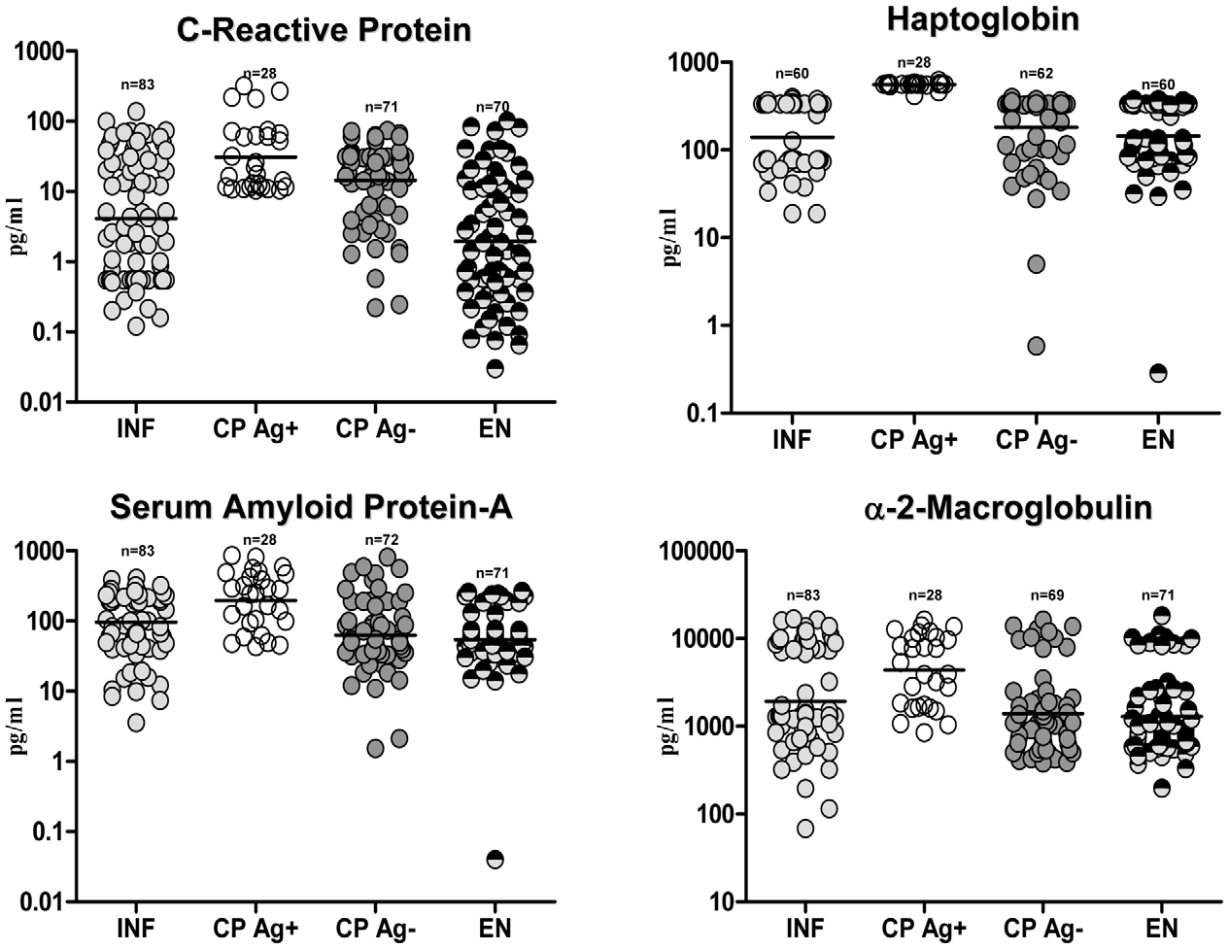
Filarial lymphedema is associated with elevated levels of acute phase proteins. Plasma levels of CRP, Haptoglobin, SAA and α-2 macroglobulin from asymptomatic infected [INF] individuals; filarial lymphedema individuals with active infection [CP Ag+]; filarial lymphedema individuals without active infection [CP Ag−] and endemic normal [EN] individuals were measured by ELISA. Data are shown as scatter plots with the bar representing the geometric mean.

To determine the association of acute phase proteins with filarial disease, we measured the plasma levels of CRP, haptoglobin, SAA, and α-2m in the four groups. As shown in figure 2, CP Ag+ had significantly higher levels of CRP (GM of 30.9 pg/ml in CP Ag+ vs. 4.11 in INF; *P*<0.0001), haptoglobin (GM of 555.9 pg/ml in CP Ag+ vs. 140.1 in INF; *P*<0.0001), SAA (GM of 196.7 pg/ml in CP Ag+ vs. 96.9 in INF; *P* = 0.0037), and α-2m (GM of 4383 pg/ml in CP Ag+ vs. 1923 pg/m in INF; *P* = 0.0003) in comparison to INF. Similarly, among those without evidence of active filarial infection (Ag−), those with CP had significantly higher levels of CRP in comparison to EN (GM of 14.5 pg/ml in CP Ag− vs.1.9 in EN; *P*<0.0001), indicating that elevated CRP levels might be more reflective of the secondary events associated with pathology than with active infection. Thus, filarial lymphedema with active infection is characterized by elevated levels of several acute phase proteins.

### CP Ag+ exhibit elevated levels of IL-1β, IL-12, and TNF-α compared with INF

**Figure 3:**
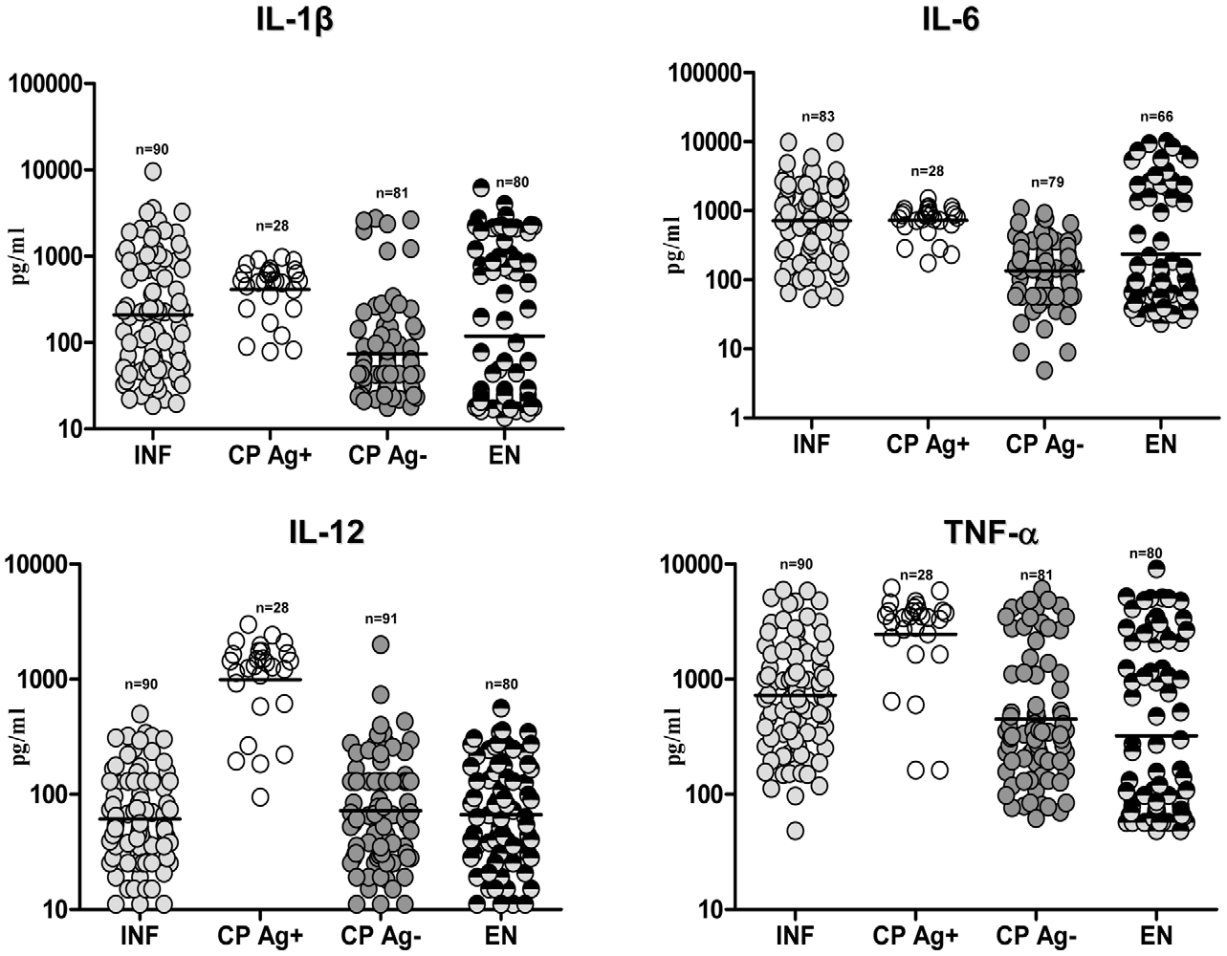
Filarial lymphedema is associated with elevated levels of inflammatory cytokines. Plasma levels of IL-1β, IL-6, IL-12 and TNF-α from asymptomatic infected [INF] individuals; filarial lymphedema individuals with active infection [CP Ag+]; filarial lymphedema individuals without active infection [CP Ag−] and endemic normal [EN] individuals were measured by ELISA. Data are shown as scatter plots with the bar representing the geometric mean.

To determine the association of inflammatory cytokines with filarial lymphedema, we measured the plasma levels of IL-1β, IL-6, IL-12, and TNF-α in the four groups of subjects. As shown in figure 3, compared with INF, those with CP Ag+ had significantly higher levels of IL-1β (GM of 410.9 pg/ml in CP Ag+ vs. 210.3 in INF; *P* = 0.0305), IL-12 (GM of 989.1 pg/ml in CP Ag+ vs. 61.2 in INF; *P*<0.0001), and TNF-α (GM of 2455 pg/ml in CP Ag+ vs. 727.2 in INF; *P*<0.0001) but not IL-6. However, no significant differences were observed in the levels of all four cytokines between those without active infection (EN and CP Ag−) irrespective of clinical status. Thus, filarial lymphedema with active infection is characterized by elevated plasma levels of inflammatory cytokines.

### Linear models on log transformed markers for all the four groups

We tested for three effects (CP effect, infection effect, and CP-by-infection interaction effect) on each of 12 markers using linear models on the log transformed responses. A significant (CP-by-infection) interaction effect meant that the geometric mean (GM) for the marker in the CP Ag+ group is significantly different from GM expected from the combined effects of CP and infection. As shown in Table 2, we observed significant effects in the models for LPS, LBP, CRP, Haptoglobin, SAA, IL-1β, IL-6, IL-12 and TNF-α. We then examined the details of the significant markers by rebuilding the linear model using only the significant (by adjusted p-value) effects. For LPS, Haptoglobin, IL-1β, and IL-12, we observed that the CP Ag+ group had significantly higher responses than the other 3 groups, while for LBP we observed that the CP Ag+ group has significantly lower responses than the other groups (see Table 3). For the 4 other markers with significant effects (CRP, SAA, IL-6, and TNF-α), we observed that CRP was significantly associated with chronic pathology (both CP Ag+ and CP Ag−); SAA and TNF- α were significantly associated with infection status (both CP Ag+ and INF); and IL-6 was associated with both pathology and the infection status (data not shown). Thus, by using robust statistical calculations, we have confirmed the association of LPS, acute phase proteins and inflammatory cytokines with filarial lymphedema with active infection. A more detailed examination of the associations is presented in the Text S1.

### Relationships between LPS/LBP levels and inflammatory cytokines in infected individuals

**Figure 4:**
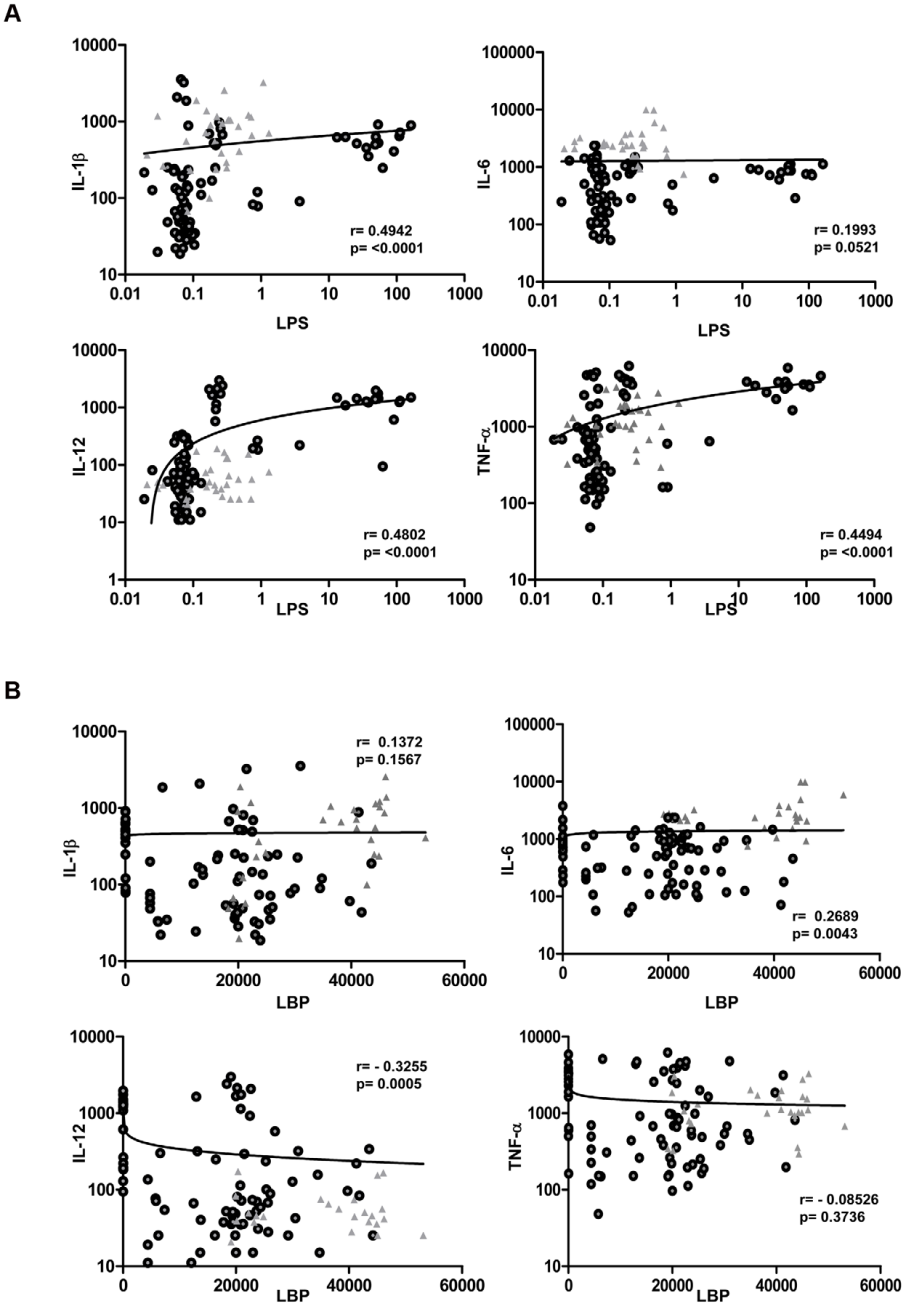
Correlation between circulating microbial products and inflammatory cytokines in filarial infected individuals. (A) Plasma levels of LPS were correlated with the levels of IL-1β, IL-6, IL-12 and TNF-α from individuals with active infection [CP Ag+ and INF (*n* = 108–112)]. (B) Plasma levels of LBP were correlated with the levels of IL-1β, IL-6, IL-12 and TNF-α from individuals with active infection [CP Ag+ and INF (*n* = 108–112)]. P and r values were calculated using the Spearman Rank correlation test. Data are shown as scatter plots with the circles representing INF and the triangles representing CP Ag+ individuals.

The relationships between the levels of LPS and/or LBP levels and plasma cytokines were next assessed (figure 4). As shown in figure 4A, levels of LPS exhibited a highly significant positive correlation with the plasma levels of IL-1β (r = 0.4942; *P*<0.001), IL-12 (r = 0.4802; *P*<0.0001), and TNF-α (r = 0.4494; *P*<0.0001) in all actively infected individuals. Conversely, LBP levels were significantly negatively correlated with the plasma levels of IL-12 (r = −0.3255; *P* = 0.0005) (figure 4B). Thus, the process by which microbial translocation occurs appears to be significantly associated with the pro-inflammatory cytokine levels in filarial infection.

**Figure 5:**
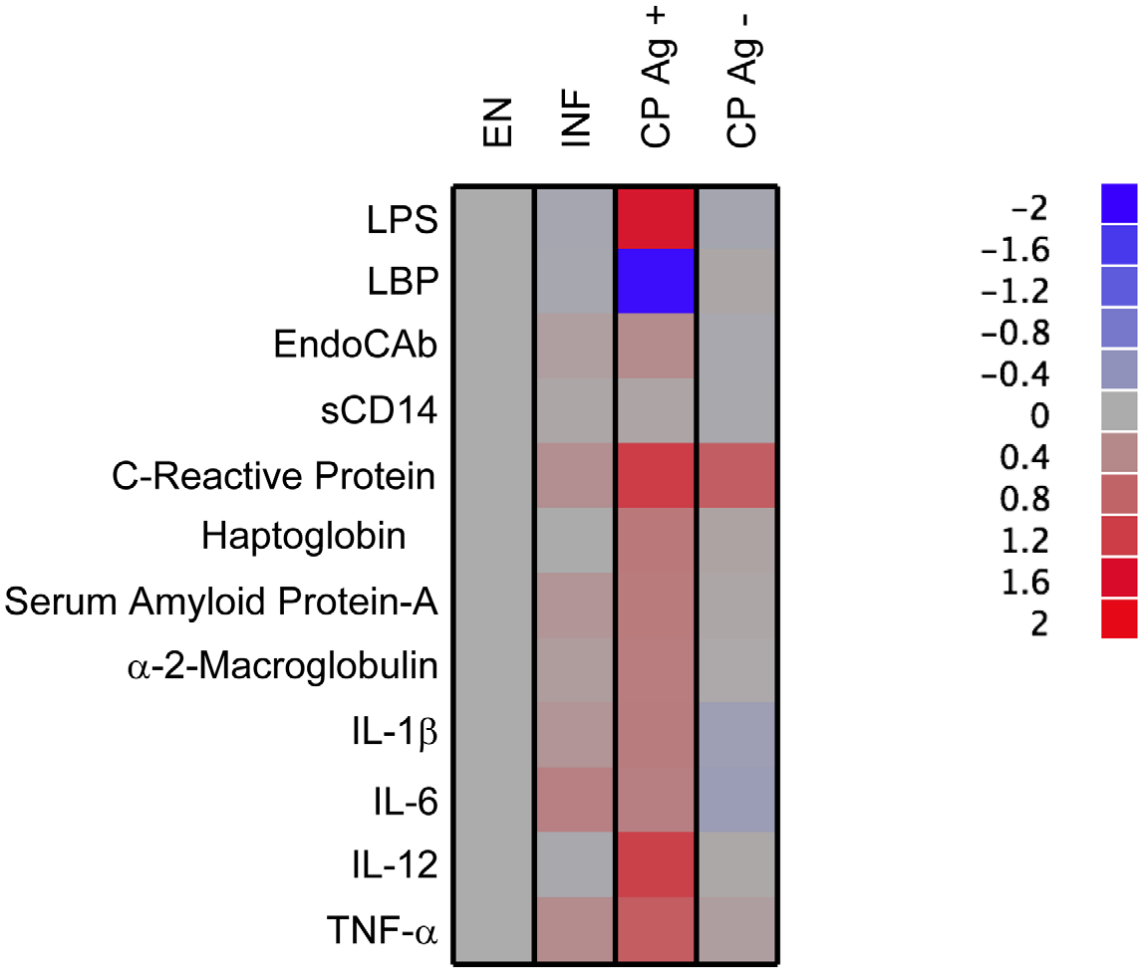
Heatmap depicting circulating microbial products, acute phase proteins and inflammatory cytokines in CP Ag+ individuals compared to EN, INF and CP Ag− individuals. Data (and scale) are log10 geometric mean fold change from EN for each of the analytes measured for each of the groups.

We also compiled the comparative analysis of all the 12 parameters in the 4 groups of individuals as a heat map, depicting the log transformed data on a scale relative to EN. As shown in figure 5, CP Ag+ individuals exhibit a distinct biomarker signature characterized by elevated levels of LPS, acute phase proteins, and certain inflammatory cytokines compared with the other 3 groups (EN, INF, and CP Ag−), again reiterating the important association of these factors with pathogenesis of filarial pathology.

## Discussion

Studies in experimental animal models suggest that intestinal injury and systemic endotoxemia are two major factors leading to morbidity in helminth infections [14], [15]. Disruption of the integrity of the intestinal epithelium and translocation of microbial products into the circulation is thought to occur in intestinal helminth infections [16]. Thus, infection with intestinal helminths is characterized by enhanced leakiness of the intestinal epithelium, mediated by activated mast cells, which can lead to the movement of bacterial LPS into the portal circulation [17], [18]. Even in non-intestinal helminth infections, such as schistosomes that reside in the mesenteric veins, damage caused by worm eggs traversing the gastrointestinal epithelium can result in systemic translocation of bacteria [14], [19]; however, the role of microbial translocation in the pathogenesis of disease in systemic helminth infections is not clear.

Lymphatic filariasis is a disease characterized by the dysfunction of lymphatics leading to severe (and often) irreversible lymphedema and elephantiasis. It has been shown that residence of adult parasites in the lymphatics leads to a cascade of events that ultimately results in tissue scarring and fibrosis [20]. Studies addressing the mechanisms underlying parasite-induced lymphatic dilatation suggest that parasite-mediated lymphatic dilatation and lymphangiogenesis might be important features in the development of pathology [7], [12]. In addition, the more severe forms of lymphedema are often associated with secondary bacterial and/or fungal infections leading to dermatolymphangioadenitis, which also contribute to the pathogenesis of disease [2]. Finally, filarial lymphedema has been shown to be associated with increased bacterial loads in the lymphatics [21], [22], [23], [24]; these damaged lymphatics could then serve as a potential nidus for bacterial translocation through the lymphatic endothelium. Thus, the predominant feature of lymphatic filarial disease is the establishment of a systemic inflammatory milieu due to both parasite-derived and host-induced inflammation.

We examined four important circulating microbial or related products in our study. LPS (a key indicator of microbial translocation) was found to be significantly elevated in CP Ag+ compared with INF but not in CP Ag− compared with EN. In addition, LPS levels were also found to be significantly elevated in the CP Ag+ compared to all the other 3 groups combined or individually. Strikingly, we observed the exactly opposite profile with LBP, the LPS binding protein commonly produced by gastrointestinal and hepatic epithelial cells in response to LPS stimulation [25]. LBP is also known to bind and transfer LPS to high-density lipoproteins to decrease the bioactivity of LPS [25] and therefore, the lower levels of LBP in CP Ag+ individuals might reflect an inability to clear LPS in circulation. Although we examined the levels of sCD14, which binds LPS and is produced by monocytes/macrophages [25], and the naturally occurring IgG antibody to the LPS core oligosaccharide (EndoCAb) [26] in all groups of subjects, we found no differences in these particular molecules. Our study therefore suggests that circulating LPS and LBP (but not sCD14 or EndoCAb) are potentially associated either with the development of pathology or function as markers for pathogenesis. While elevated levels of LPS in CP Ag+ compared with INF could potentially be attributed to presence of secondary bacterial infection, the elevated immune activation observed in chronic pathology patients with active infection suggests that the interaction between filarial infection and pathology is a major contributor to microbial translocation, fueling systemic immune activation. Interestingly, our findings are similar to findings reported in other infectious diseases also characterized by systemic immune activation, including HIV [27], [28], hepatitis B and C [29], and schistosomiasis [19], [30].

Acute phase proteins derive primarily from the liver, and plasma concentrations are felt to be a reflection of the response to pro-inflammatory cytokines [31]. Measurement of acute phase proteins is of clinical importance in determining the presence and extent of inflammatory tissue damage as well as in providing diagnostic and prognostic information [32], [33]. Moreover, circulating microbial products are well known inducers of acute phase proteins, with SAA and haptoglobin known to be markedly elevated following challenge with LPS [34]. Elevated levels of CRP have been reported in lymphatic filarial disease [35], but other acute phase proteins have not been examined. In the present study, we confirmed that CRP levels are indeed elevated in actively infected patients with chronic lymphedema compared with the asymptomatic group, but we also found that haptoglobin, SAA, and α-2m are also elevated. Upon, further analysis, only haptoglobin was observed to be significantly associated with filarial-infection with pathology, while SAA was significantly associated with filarial infection per se (both CP Ag+ and INF). Interestingly, CRP levels were significantly elevated even in those patients with chronic lymphedema without active infection, indicating that CRP is probably a nonspecific marker of inflammation in filarial disease, whereas haptoglobin might serve as a more accurate biomarker of filarial infection-driven pathology.

Although persistent and progressive inflammation is postulated to be a hallmark of lymphatic filarial disease, very few studies have actually examined the levels of inflammatory cytokines or chemokines in the circulation of infected or diseased individuals. Previous reports have suggested that IL-6 and IL-8 are morbidity markers in acute and chronic disease [36], while IL-6 and TNF-α are involved in the pathogenesis of adverse reactions following treatment [37]. Our examination of cytokine expression levels in the four groups of individuals reveals that IL-1β and IL-12 are significantly associated with overt pathology in actively infected individuals. Conversely, TNF-α was associated significantly with groups having active infection (CP Ag+ and INF) indicating a possible association with filarial infection rather than pathology alone. Because inflammatory cytokines are intricately linked to induction of both circulating microbial products and acute phase proteins, we also examined their interrelationship in the CP Ag+ population. Detection of microbial invasion by cells of the innate immune system usually results in increased production of pro-inflammatory cytokines such as IL-1β, IL-6, IL-12, and TNF-α [11]. Studies in HIV infection reveal a direct association between levels of microbial translocation markers such as LPS and the inflammatory cytokines [27], [28]. In agreement with such studies, our examination of filaria-infected individuals also reveals a significantly positive association between LPS and pro-inflammatory cytokines in filaria-infected-individuals. Our study clearly implicates an association for LPS, the acute phase proteins, and several of the pro-inflammatory cytokines with filaria-induced lymphatic pathology.

Investigations into filarial disease pathogenesis have implicated host pathways in disease progression. In particular, dysregulated inflammatory responses and lymphatic dysfunction are thought to be central processes in severe filarial pathology [7], [12]. Our study reveals novel insights into the pathogenesis of lymphatic filarial dysfunction, despite some minor limitations. Since DEC had been administered only to the CP Ag− group and the presence of other parasitic infections not examined, the effect of treatment with DEC as well as the influence of other parasitic infections could not be ascertained in this study.

Another minor limitation of the study was that plasma levels of inflammatory markers—such as circulating microbial products, acute phase proteins, and cytokines—are relatively nonspecific and may be influenced by short half-life, nonspecific induction, and plasma levels not reflecting biologic activity. Notwithstanding these limitations, plasma levels of some of these same biomarkers have yielded important insights in the diagnosis and/or prognosis of various infectious diseases and cancers [10], [38], [39].

Our study clearly identifies a signature set of biomarkers that serves to indicate filarial infection-driven morbidity associated with a persistent and progressive inflammatory milieu. While requiring validation in future studies, these results point to potential prognostic indicators of severe filarial disease.

## Supporting Information

Text S1 Linear models on log transformed data on all the markers in all four groups (CP Ag+, INF, CP Ag− and EN).
(DOC)

## References

[1] Nutman TB, Kumaraswami V (2001). Regulation of the immune response in lymphatic filariasis: perspectives on acute and chronic infection with *Wuchereria bancrofti* in South India.. Parasite Immunol.

[2] Dreyer G, Noroes J, Figueredo-Silva J, Piessens WF (2000). Pathogenesis of lymphatic disease in bancroftian filariasis: a clinical perspective.. Parasitol Today.

[3] de Almeida AB, Freedman DO (1999). Epidemiology and immunopathology of bancroftian filariasis.. Microbes Infect.

[4] Steel C, Ottesen EA, Weller PF, Nutman TB (2001). Worm burden and host responsiveness in *Wuchereria bancrofti* infection: use of antigen detection to refine earlier assessments from the South Pacific.. Am J Trop Med Hyg.

[5] Babu S, Anuradha R, Pavan Kumar N, George PJ, Kumaraswami V (2011). Filarial Lymphatic Pathology Reflects Augmented TLR-mediated, MAPK-mediated Pro-inflammatory Cytokine Production.. Infect Immun.

[6] Babu S, Bhat SQ, Pavan Kumar N, Lipira AB, Kumar S (2009). Filarial lymphedema is characterized by antigen-specific Th1 and th17 proinflammatory responses and a lack of regulatory T cells.. PLoS Negl Trop Dis.

[7] Pfarr KM, Debrah AY, Specht S, Hoerauf A (2009). Filariasis and lymphoedema.. Parasite Immunol.

[8] Freedman DO, Plier DA, de Almeida A, Miranda J, Braga C (1999). Biased TCR repertoire in infiltrating lesional T cells in human Bancroftian filariasis.. J Immunol.

[9] Lawrence RA, Devaney E (2001). Lymphatic filariasis: parallels between the immunology of infection in humans and mice.. Parasite Immunol.

[10] Nixon DE, Landay AL (2010). Biomarkers of immune dysfunction in HIV.. Curr Opin HIV AIDS.

[11] Medzhitov R (2007). Recognition of microorganisms and activation of the immune response.. Nature.

[12] Bennuru S, Nutman TB (2009). Lymphatics in human lymphatic filariasis: in vitro models of parasite-induced lymphatic remodeling.. Lymphat Res Biol.

[13] Chakera A, Lucas A, Lucas M (2011). Surrogate markers of infection: interrogation of the immune system.. Biomark Med.

[14] Herbert DR, Holscher C, Mohrs M, Arendse B, Schwegmann A (2004). Alternative macrophage activation is essential for survival during schistosomiasis and downmodulates T helper 1 responses and immunopathology.. Immunity.

[15] Leeto M, Herbert DR, Marillier R, Schwegmann A, Fick L (2006). TH1-dominant granulomatous pathology does not inhibit fibrosis or cause lethality during murine schistosomiasis.. Am J Pathol.

[16] Robinson MW, Donnelly S, Hutchinson AT, To J, Taylor NL (2011). A family of helminth molecules that modulate innate cell responses via molecular mimicry of host antimicrobial peptides.. PLoS Pathog.

[17] Farid AS, Jimi F, Inagaki-Ohara K, Horii Y (2008). Increased intestinal endotoxin absorption during enteric nematode but not protozoal infections through a mast cell-mediated mechanism.. Shock.

[18] McDermott JR, Bartram RE, Knight PA, Miller HR, Garrod DR (2003). Mast cells disrupt epithelial barrier function during enteric nematode infection.. Proc Natl Acad Sci U S A.

[19] Ferraz AA, Campos JM, Junior JG, De Albuquerque AC, Ferraz EM (2005). Gut bacterial translocation and postoperative infections: a prospective study in schistosomotic patients.. Surg Infect (Larchmt).

[20] Figueredo-Silva J, Noroes J, Cedenho A, Dreyer G (2002). The histopathology of bancroftian filariasis revisited: the role of the adult worm in the lymphatic-vessel disease.. Ann Trop Med Parasitol.

[21] Olszewski W, Jamal S (1994). Skin bacterial factor in progression of filarial lymphedema.. Lymphology.

[22] Olszewski WL, Jamal S, Manokaran G, Pani S, Kumaraswami V (1997). Bacteriologic studies of skin, tissue fluid, lymph, and lymph nodes in patients with filarial lymphedema.. Am J Trop Med Hyg.

[23] Olszewski WL, Jamal S, Manokaran G, Pani S, Kumaraswami V (1999). Bacteriological studies of blood, tissue fluid, lymph and lymph nodes in patients with acute dermatolymphangioadenitis (DLA) in course of ‘filarial’ lymphedema.. Acta Trop.

[24] Swoboda-Kopec E, Luczak M, Lukomska B, Olszewski WL, Jamal S (1999). [Bacterial infections of skin and soft tissues in filariasis].. Med Dosw Mikrobiol.

[25] Kitchens RL, Thompson PA (2005). Modulatory effects of sCD14 and LBP on LPS-host cell interactions.. J Endotoxin Res.

[26] Cohen IR, Norins LC (1966). Natural human antibodies to gram-negative bacteria: immunoglobulins G, A, and M.. Science.

[27] Brenchley JM, Price DA, Schacker TW, Asher TE, Silvestri G (2006). Microbial translocation is a cause of systemic immune activation in chronic HIV infection.. Nat Med.

[28] Nowroozalizadeh S, Mansson F, da Silva Z, Repits J, Dabo B (2010). Microbial translocation correlates with the severity of both HIV-1 and HIV-2 infections.. J Infect Dis.

[29] Sandler NG, Koh C, Roque A, Eccleston JL, Siegel RB (2011). Host Response to Translocated Microbial Products Predicts Outcomes of Patients With HBV or HCV Infection.. Gastroenterology.

[30] Onguru D, Liang Y, Griffith Q, Nikolajczyk B, Mwinzi P (2011). Human schistosomiasis is associated with endotoxemia and Toll-like receptor 2- and 4-bearing B cells.. Am J Trop Med Hyg.

[31] Baumann H, Gauldie J (1990). Regulation of hepatic acute phase plasma protein genes by hepatocyte stimulating factors and other mediators of inflammation.. Mol Biol Med.

[32] Johnson HL, Chiou CC, Cho CT (1999). Applications of acute phase reactants in infectious diseases.. J Microbiol Immunol Infect.

[33] Peracaula R, Sarrats A, Rudd PM (2010). Liver proteins as sensor of human malignancies and inflammation.. Proteomics Clin Appl.

[34] Levels JH, Geurts P, Karlsson H, Maree R, Ljunggren S (2011). High-density lipoprotein proteome dynamics in human endotoxemia.. Proteome Sci.

[35] Lal RB, Dhawan RR, Ramzy RM, Farris RM, Gad AA (1991). C-reactive protein in patients with lymphatic filariasis: increased expression on lymphocytes in chronic lymphatic obstruction.. J Clin Immunol.

[36] Satapathy AK, Sartono E, Sahoo PK, Dentener MA, Michael E (2006). Human bancroftian filariasis: immunological markers of morbidity and infection.. Microbes Infect.

[37] Turner PF, Rockett KA, Ottesen EA, Francis H, Awadzi K (1994). Interleukin-6 and tumor necrosis factor in the pathogenesis of adverse reactions after treatment of lymphatic filariasis and onchocerciasis.. J Infect Dis.

[38] Pang WW, Abdul-Rahman PS, Wan-Ibrahim WI, Hashim OH (2010). Can the acute phase reactant proteins be used as cancer biomarkers?. Int J Biol Markers.

[39] Walzl G, Ronacher K, Hanekom W, Scriba TJ, Zumla A (2011). Immunological biomarkers of tuberculosis.. Nat Rev Immunol.

