# Supplementary material for: Circulating Microbial Products and Acute Phase Proteins as Markers of Pathogenesis in Lymphatic Filarial Disease
Source: PLoS Pathog. 2012 Jun 7;8(6):e1002749. doi: 10.1371/journal.ppat.1002749 (PMC3369944; doi:10.1371/journal.ppat.1002749)
Supplement: Text S1 — Linear models on log transformed data on all the markers in all four groups (CP Ag+, INF, CP Ag− and EN). (DOC) [file ppat.1002749.s001.doc]

Circulating Microbial Products and Acute Phase Proteins as Markers of Pathogenesis in Lymphatic Filarial Disease

R. Anuradha,^1^ P. Jovvian George,^1^ N. Pavan Kumar,^1^ Michael P. Fay,^3^ V. Kumaraswami,^4^ Thomas B. Nutman,^5^ and Subash Babu,^1,2^,

^1^National Institutes of Health—International Center for Excellence in Research, Chennai, India, ^2^SAIC‑Frederick, Inc., NCI‑Frederick, Frederick, Maryland, USA, ^3^Biostatistics Research Branch, National Institute of Allergy and Infectious Diseases, National Institutes of Health, Bethesda, Maryland, USA, ^4^Tuberculosis Research Center, Chennai, India, and ^5^Laboratory of Parasitic Diseases, National Institute of Allergy and Infectious Diseases, National Institutes of Health, Bethesda, Maryland, USA

Text S1:

***Linear models on log transformed markers for all the four groups.***

We tested for three effects (CP effect, infection effect, and CP-by-infection interaction effect) on each of 12 markers using linear models on the log transformed responses. A significant (CP-by-infection) interaction effect meant that the geometric mean (GM) for the marker in the CP Ag+ group is significantly different from GM expected from the combined effects of CP and infection. Table 2 shows the p-values for the 36 tests adjusted for multiple comparison, so p_adjusted_<0.05 denotes that there is less than a 5% chance we would have seen something that extreme or more extreme in any of the 36 tests given that all the effects were caused by pure chance. The sample sizes are different because each marker was not measured on every subject.

We then examined the details of the significant markers by rebuilding the linear model using only the significant (by adjusted p-value) effects. First we considered markers only significant at the CP-by-infection interaction. For those models, the interaction effect could be interpreted as comparing the CP Ag+ group to the other 3 groups combined. For LPS, Haptoglobin, IL-1β, and IL-12, we observed that the CP Ag+ group had significantly higher responses than the other 3 groups, while for LBP we observed that the CP Ag+ group has significantly lower responses than the other groups (see Table 3). We also noted that although the LBP effects were due to extreme effects in only a subset of the CP Ag+ subjects (Figure 1), the statistical tests were nonetheless valid and the effect represented the average fold-change.

For the 4 other markers with significant effects (CRP, SAA, IL-6, and TNF-α), we again rebuilt models including terms only if they were significant. Groups with CP (CP Ag+ and CP Ag-) exhibited 6.15 (95% CI 4.05, 9.34) times larger GM for CRP compared to groups without CP. On the other hand, groups with infection (CP Ag+ and INF) exhibited 1.98 (95% CI 1.51, 2.60) times larger GM for SAA, and 2.55 (95% CI 1.86, 3.49) times larger GM for TNF-α compared to groups without infection. For IL-6, we observed significant effects for both CP (Fold-change=1.42, 95% CI 1.09, 1.85) and infection (fold-change=7.04, 95% CI 5.38, 9.21). In addition, the CP Ag+ group exhibited 10.0 (=1.42*7.04) times larger GM for IL-6 than the EN group.

We also observed that repeating the analyses after adjusting for age yielded very similar results (results not shown). Finally, we observed from the LPB data (Figure 1) that there were 18 subjects with very low LBP (below 100) in the CP Ag+ group, while the other 10 subjects appear to have approximately normal levels of LBP (range 12961 - 26892). We therefore measured and compared the GM for this group compared to the others; however, since this group was defined after examining the data, we presented these fold-changes as descriptive statistics only (i.e., without p-values or confidence intervals). The GM for markers of these 18 subjects compared with all the other subjects were much larger for LPS (fold-change=199.1), Haptoglobin (fold-change=3.34), IL-1β (fold-change=2.78) and IL-12 (fold-change=10.18). This group (of course) had much smaller LBP (fold-change=0.0012).
